# Optimizing Reconstruction Parameters for Detecting Peripheral In-Stent Restenosis with Photon-Counting Detector CT: A Phantom Study

**DOI:** 10.3390/diagnostics16091253

**Published:** 2026-04-22

**Authors:** Yiheng Tan, Joost F. Hop, Magdalena Dobrolinska, Xinlin Zheng, Evie J. I. Hoeijmakers, Jean-Paul P. M. de Vries, Marcel J. W. Greuter, Reinoud P. H. Bokkers

**Affiliations:** 1 Department of Radiology, University Medical Centre Groningen, University of Groningen, 9713 GZ Groningen, The Netherlands; y.tan@umcg.nl (Y.T.);; 2 Department of Nuclear Medicine and Molecular Imaging, University Medical Centre Groningen, University of Groningen, 9713 GZ Groningen, The Netherlands; 3 Division of Cardiology and Structural Heart Diseases, Medical University of Silesia, 40-514 Katowice, Poland; 4 Department of Radiology and Nuclear Medicine, Maastricht University Medical Centre+, 6202 AZ Maastricht, The Netherlands; 5 CARIM School for Cardiovascular Diseases, Maastricht University, 6229 ER Maastricht, The Netherlands; 6 Department of Surgery, Division of Vascular Surgery, University Medical Centre Groningen, University of Groningen, 9713 GZ Groningen, The Netherlands

**Keywords:** computed tomography, photon-counting detector CT, in-stent restenosis, peripheral arterial disease, virtual monoenergetic image

## Abstract

**Background/Objectives**: To determine the optimal reconstruction parameters for accurate visualization of peripheral in-stent restenosis using photon-counting detector CT (PCD-CT), and to evaluate its potential advantages over energy-integrated detector CT (EID-CT). **Methods**: Endovascular peripheral stents with varying degrees of in-stent restenosis were scanned in a custom-made phantom using EID-CT (Somatom Force) and PCD-CT (Naeotom Alpha) under clinical acquisition protocols. EID-CT images were reconstructed with Bv40 and Bv59 kernels at 512 matrices. PCD-CT data were acquired in standard-resolution (SR) and ultra-high-resolution (UHR) modes. In both modes, images were reconstructed with multiple kernels (Bv40, Bv56 and Bv72) and matrix sizes (512 and 1024 matrix). In SR mode, additional virtual monoenergetic images (40–100 keV) were generated, while UHR mode included only polychromatic reconstructions. Quantitative image quality (noise, contrast, contrast-to-noise ratio [CNR]) was measured, and two blinded readers performed qualitative assessments of restenosis visualization. **Results**: PCD-CT with SR mode at VMI 40 keV achieved the highest image contrast and CNR, significantly outperforming EID-CT and PCD-CT_UHR_ under matched conditions (all *p* < 0.05). The sharper reconstruction kernel further enhanced the image contrast and improved subjective visualization despite increased image noise. Both readers ranked PCD-CT_SR-Bv72-40keV_ at 1024 matrix highest for detecting all degrees of restenosis, with excellent inter-reader agreement (ρ > 0.80). **Conclusions**: PCD-CT in SR mode at VMI 40 keV, specifically using the Bv72 kernel with a 1024 matrix, optimizes the visualization of peripheral in-stent restenosis. Compared to EID-CT, PCD-CT provides superior image quality and detectability of restenosis.

## 1. Introduction

Stent implantation often plays a key role in preventing arterial recoil and re-occlusion [1], but in-stent restenosis remains a major cause of treatment failure, with reported rates ranging from about 5% in the iliac arteries to more than 50% in infrapopliteal segments [2,3].

Computed tomography angiography (CTA) is a widely available, non-invasive tool for evaluating vascular patency and stent integrity after endovascular treatment. It is frequently used in patients with peripheral arterial disease to assess vessel patency and detect restenosis. CTA provides high spatial and temporal resolution, large anatomical coverage, and three-dimensional visualization of the arterial tree. However, conventional energy-integrating detector CT (EID-CT) remains limited by metal-related blooming artifacts and partial volume effects, which obscure the in-stent lumen and reduce diagnostic confidence in assessing restenosis.

Photon-counting detector computed tomography (PCD-CT) is an emerging imaging technology that offers enhanced spatial resolution, reduced metallic artifacts, and improved contrast-to-noise ratio by directly converting X-ray photons into electrical signals [4]. Compared to EID-CT, PCD-CT enables ultra-high-resolution up to 0.2 × 0.2 mm. Additionally, the virtual monoenergetic images of PCD-CT enhances the visualization of iodinated contrast, thereby potentially improving the detection of in-stent restenosis [4,5]. Although recent studies have demonstrated superior image quality of PCD-CT over EID-CT for in-stent restenosis detection [6,7], the optimal reconstruction parameters that balance noise, contrast, and sharpness to achieve the best visualization of in-stent restenosis remain unclear.

This study aimed to determine the optimal reconstruction parameters for accurate visualization of peripheral in-stent restenosis using PCD-CT, and to evaluate its potential advantages over EID-CT.

## 2. Materials and Methods

### 2.1. Phantom Setup

**Figure 1:**
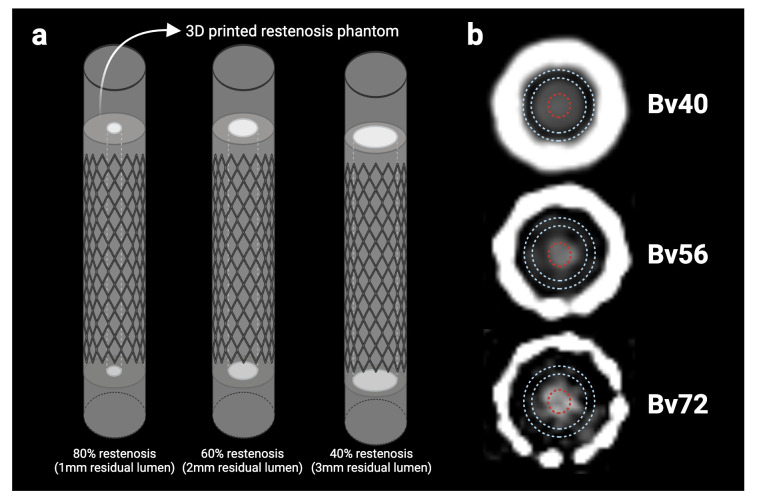
Illustration of stent restenosis models (**a**). Two regions of interest (ROIs) were placed within the restenosis plaque (blue) and the residual lumen (red) (**b**).

A custom-built in-stent restenosis phantom was developed to allow systematic comparison between PCD- and EID-CT. Three peripheral stents (Cook Medical, Limerick, Ireland) with a diameter of 5 mm were used. To simulate hypodense stenoses, three cylindrical models were fabricated using a 3D printer with clear resin (Formlabs Inc., Somerville, MA, USA), with a CT number of approximately 100 Hounsfield Units (HU) at 120 kVp. Each cylindrical stenosis model included a central lumen with a diameter of 1 mm, 2 mm, or 3 mm, representing 80%, 60%, and 40% restenosis, respectively. These stenosis models were inserted into the stents and connected to 5 mm diameter silicone tubes, which were then filled with iodinated contrast agent (Iomeron 350 mg/mL, Bracco Imaging S.p.A., Milan, Italy) titrated to approximately 400 HU at 120 kVp (Figure 1a). Both ends of the tubes were sealed, and the entire model was placed into the water-filled holes of a 16 cm diameter CTDI phantom (PTW Freiburg GmbH, Freiburg, Germany) to simulate in vivo imaging conditions.

### 2.2. CT Acquisition

Imaging was performed using a first generation PCD-CT system (Naeotom Alpha, Software version VB10, Siemens Healthineers, Forchheim, Germany) and a third generation dual-source EID-CT system (Somatom Force, Siemens Healthineers). Acquisition parameters were based on clinical CTA protocols (Table 1).

The PCD-CT was operated in two resolution modes: an ultra-high-resolution mode (PCD-CT_UHR_) with 120 × 0.2 mm collimation, image quality (IQ) level of 80, rotation time of 0.5 s, and a pitch of 0.85; and a standard-resolution mode (PCD-CT_SR_) with 144 × 0.4 mm collimation, IQ level of 100, rotation time of 0.5 s, and a pitch of 0.8. Both modes were acquired at 120 kVp tube voltage.

The EID-CT scanner was operated at 120 kVp and reference mAs of 90, with 192 × 0.6 mm collimation, a rotation time of 0.5 s, and a pitch of 0.6.

### 2.3. Image Reconstruction

In PCD-CT_UHR_ mode, images were reconstructed with matrix sizes of 1024 × 1024 and 512 × 512, using a slice thickness of 0.2 mm and an increment of 0.2 mm. PCD-CT_UHR_ datasets were reconstructed as polychromatic images (T3D) because VMIs were not available at 0.2 mm slice thickness. In PCD-CT_SR_ mode, reconstruction was also performed with both matrix sizes, with a slice thickness of 0.4 mm and a slice increment of 0.3 mm. All PCD-CT_SR_ datasets were reconstructed at VMI energy levels of 40, 55, 70, 85, and 100 keV, as well as T3D images. Three reconstruction kernels were applied in both PCD-CT modes: soft (Bv40), medium-sharp (Bv56), and sharp (Bv72). A quantum iterative reconstruction algorithm at a strength level of three (QIR 3) was applied.

For EID-CT, images were reconstructed with a slice thickness of 0.5 mm and a slice increment of 0.3 mm, using a 512 × 512 matrix. Soft (Bv40) and medium-sharp (Bv59) kernels were used.

### 2.4. Image Assessment

#### 2.4.1. Quantitative Image Assessment

The image noise level, image contrast, and contrast-to-noise ratio (CNR) were evaluated. To assess image noise, a region of interest (ROI) was placed within the CTDI phantom on each slice containing the stents. Noise was defined as the standard deviation of HU within this ROI. All reconstructed images of moderate (60%) restenosis were analyzed for image contrast and CNR using an in-house algorithm. First, the stent boundary coordinates were defined, and the stents were cropped from the image stack accordingly across all reconstructions. The center of each stent was identified, and two ROIs were generated on each slice: a circular ROI placed within the residual lumen and a concentric ring ROI placed in the restenosis plaque (Figure 1b). The mean HU values of the lumen and plaque were recorded for each slice, and the image contrast and CNR were calculated as follows:

Image contrast = HU_lumen_ − HU_plaque_

CNR = (HU_lumen_ − HU_plaque_)/Image Noise

#### 2.4.2. Qualitative Image Assessment

EID-CT, PCD-CT_UHR_, and PCD-CT_SR_ at VMI 40 keV were used for subjective assessments. Two radiologists, with ≥5 years of experience in vascular imaging, independently assessed all images of the three restenosis degrees using pairwise comparison software (verison 1.0) [8]. The software separately loaded the 14 reconstructed datasets for each of the three degrees of stenosis. For each stenosis severity, pairs of images were randomly displayed side by side, and the blinded radiologists were asked to select the dataset offering superior visualization of in-stent restenosis. The radiologists were blinded to all acquisition and reconstruction parameters. All images were displayed in the longitudinal orientation of the stent, with the window width and level freely adjustable during evaluation. Through a series of comparisons, a ranking of restenosis visibility was obtained for all datasets.

### 2.5. Statistical Analysis

The Shapiro–Wilk test was used to assess normality of data distribution. Variables with normal distributions were reported as mean ± standard deviation (SD), whereas those with non-normal distributions or categorical data were presented as median and interquartile range (IQR). Objective image quality was assessed using one-way analysis of variance (ANOVA), followed by Tukey’s HSD test for post hoc pairwise comparisons. Subjective image quality rankings were assessed using Friedman test, with Dunn’s test for post hoc analysis. Comparisons of quantitative and qualitative image quality between the two matrix sizes were performed using paired t-test and Wilcoxon signed-rank test, respectively. Spearman’s rank correlation (ρ) was used to assess inter-reader agreement between two radiologists in the subjective assessment. Spearman’s rho values were interpreted as follows: <0.20, poor agreement; 0.20–0.40, fair agreement; 0.40–0.60, moderate agreement; 0.60–0.80, good agreement; and >0.80, excellent agreement. All statistical analyses were performed in Python (version 3.10.9) at a significance level of *p* < 0.05.

## 3. Results

### 3.1. Quantitative Image Analysis

**Figure 2:**
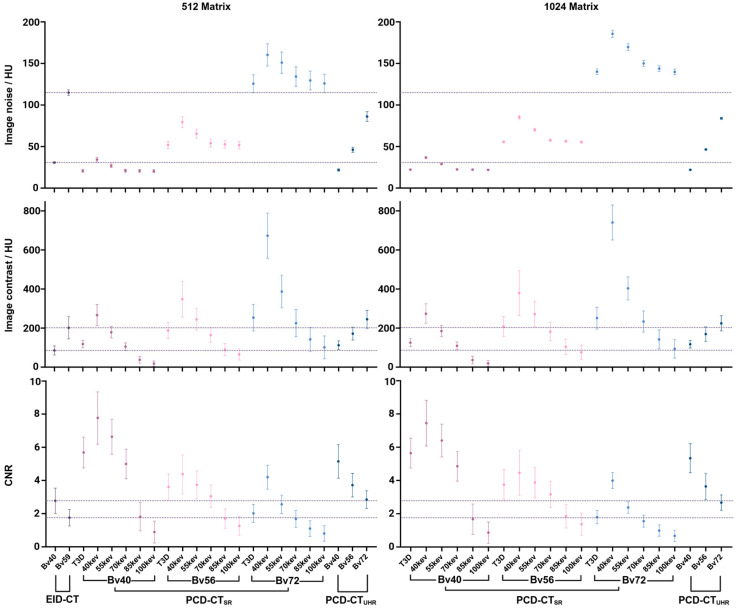
Quantitative assessment results in noise, image contrast, and CNR. The dash lines represent the mean image quality value of EID-CT_Bv40_ and EID-CT_Bv59_.

Across all scanners, image noise increased with sharper reconstruction kernels (all *p* < 0.05) (Figure 2, Table 2). Within soft kernel reconstructions (Bv40), EID-CT showed slightly lower noise than PCD-CT_SR-40keV_ but higher noise than PCD-CT_UHR_ and higher keV levels in PCD-CT_SR_ (all *p* < 0.05). In PCD-CT_SR_, VMI 40 keV produced the highest image noise, while it decreased with incremental keV levels.

Image contrast rose markedly with increasing kernel sharpness on both CT systems (all *p* < 0.05). Higher keV levels in PCD-CT_SR_ showed lower image contrast. For example, the image contrast of PCD-CT_SR-Bv72-40keV_ at 1024 matrix was 740 ± 89 HU, while it dropped to 93 ± 47 HU at PCD-CT_SR-Bv72-100keV_. Similarly, within each kernel, CNR decreased significantly with increasing keV levels (all *p* < 0.05). The CNR of PCD-CT_SR-Bv72_ under 1024 matrix dropped from 3.99 ± 0.48 at VMI 40 keV to 0.67 ± 0.34 at VMI 100 keV. However, because sharper kernels increased image noise, CNR declined as kernel sharpness increased (all *p* < 0.05). Among all reconstructions, PCD-CT with SR mode at VMI 40 keV achieved the highest image contrast and CNR, significantly outperforming EID-CT and PCD-CT_UHR_ under matched conditions (all *p* < 0.05).

### 3.2. Qualitative Image Analysis

**Figure 3:**
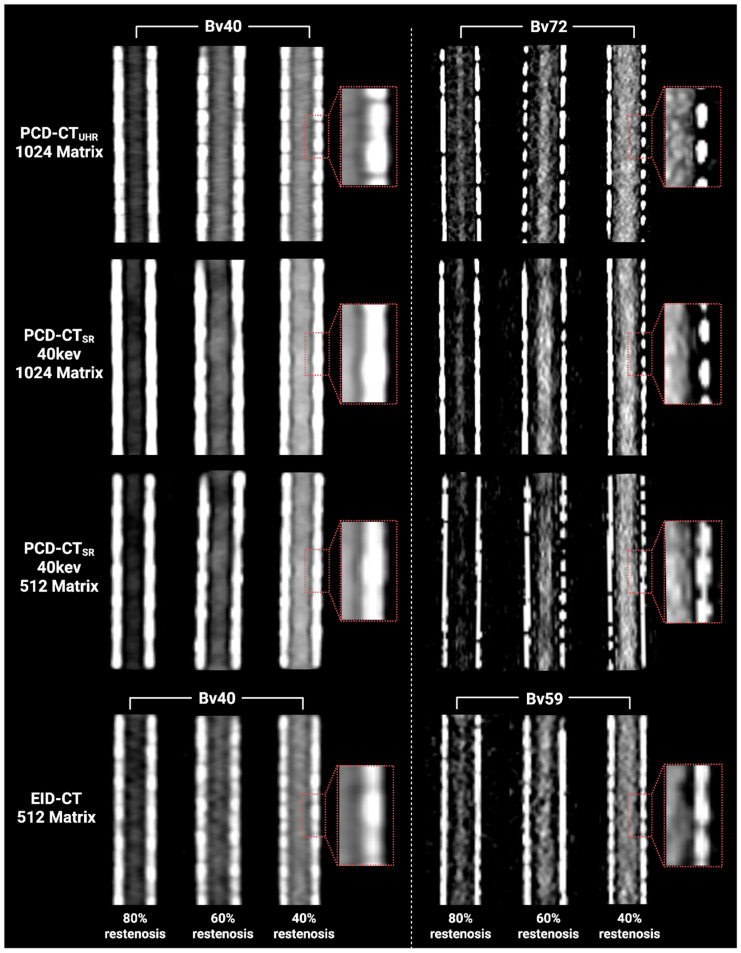
Visualization of in-stent restenosis across different CT scanners and reconstruction kernels. Sharper kernels showed better restenosis visibility on both CT systems. PCD-CT_SR-Bv72-40keV_ further enhanced the restenosis visibility, especially for 40% and 80% restenosis. The red dashed boxes indicate magnified views of the stent strut.

**Figure 4:**
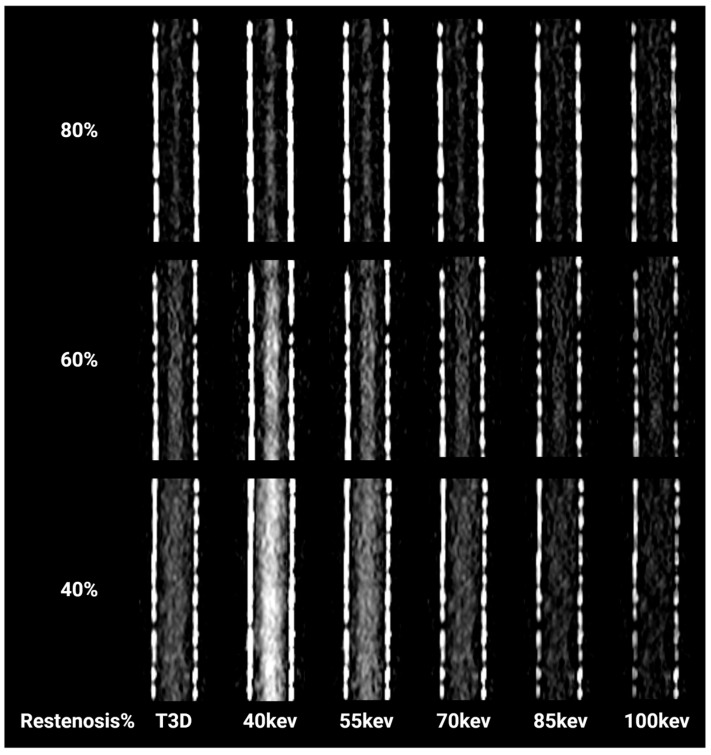
Loss of iodine contrast in VMIs from low keV to high keV in PCD-CT_SR-Bv72_. In total, 40 keV showed the highest image contrast that the other VMIs.

**Figure 5:**
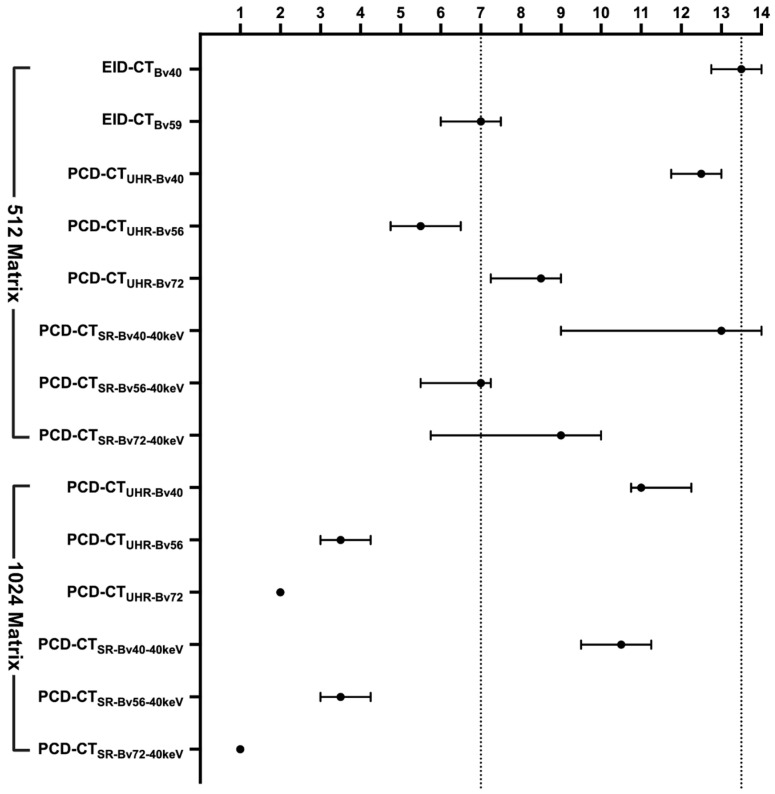
Subjective image quality ratings (median and interquartile range) for different reconstruction protocols with scores ranging from 1 (highest image quality) to 14 (lowest image quality). The horizontal axis represents the ratings, and the vertical axis represents different reconstruction parameters. For each protocol, the subjective score is shown as the median with interquartile range. The dash lines represent the median image quality rank of EID-CT_Bv40_ and EID-CT_Bv59_.

Figure 3 and Figure 4 illustrate qualitative differences in in-stent restenosis visualization across protocols. Both readers ranked PCD-CT_SR-Bv72-40 keV_ at 1024 matrix highest for all restenosis severities, followed by PCD-CT_UHR-Bv72_ at 1024 matrix (Figure 5). All degrees of restenosis were difficult to detect when using the Bv40 kernel on both CT systems.

For both PCD-CT and EID-CT, the medium-sharp and sharp kernels were rated significantly superior to the soft kernel at both matrix sizes (all *p* < 0.05). In PCD-CT, increasing the matrix size significantly improved subjective image quality for the medium-sharp and sharp kernels (all *p* < 0.05), whereas no significant improvement was observed for the Bv40 kernel. Compared to EID-CT, PCD-CT only showed a significant improvement in subjective rating when using the 1024 matrix with medium or sharp reconstruction kernels.

The inter-reader agreement for 80% stenosis (ρ = 0.956), 60% stenosis (ρ = 0.815), and 40% stenosis (ρ = 0.903) was excellent (all *p* < 0.05).

## 4. Discussion

This study demonstrated that photon-counting CT (PCD-CT) in standard-resolution (SR) mode with a sharp reconstruction kernel at VMI 40 keV provides superior image contrast and visibility of in-stent restenosis compared with both PCD-CT ultra-high-resolution (UHR) mode and a conventional energy-integrating detector CT (EID-CT). Sharper reconstruction kernels improved edge definition and restenosis delineation, while lower keV levels enhanced iodine contrast despite increased image noise.

Previous studies have reported improved stent visualization with PCD-CT mainly due to reduced blooming artifacts and higher spatial resolution [7,9,10,11]. However, most prior work has focused on detector performance or UHR acquisition without systematically assessing reconstruction parameters or energy-dependent effects. For instance, Szilveszter et al. [12] and Dachs et al. [7] showed that UHR images significantly enhanced the detection of in-stent restenosis in coronary and peripheral stents, respectively. However, VMIs were not included in their analyses. In contrast, Bratke et al. [6] and Michael et al. [13] evaluated the effect of different keV levels on in-stent restenosis detection and reported that sharp reconstruction kernel combined with low keV VMIs could improve image quality. Nevertheless, these studies were limited to 50% stenosis models. Mild and severe stenoses are often more challenging to detect due to blooming artifacts and extremely small residual lumens. The present study extends these findings by systematically identifying an optimal reconstruction parameter that combines the advantages of spectral information and high spatial resolution across varying degrees of stent stenosis.

The observed superiority of VMI 40 keV can be explained by increased photoelectric absorption near the iodine K-edge (33.2 keV), which boosts the contrast between the contrast-filled lumen and surrounding restenosis tissue [14]. Although image noise increased significantly at VMI 40 keV, the higher image contrast allows the use of a wider window setting, making the noise less perceptible [13]. However, it should be noted that VMI 40 keV may suffer from excessively high image noise in patients with large body habitus, potentially compromising diagnostic performance. The present study was limited to lower extremity stents. In addition, to ensure the accuracy of spectral analysis, we employed clear resin as the simulated restenosis material. This material exhibits HU values close to those of real soft tissue, with relatively stable attenuation across different keV levels [15].

Higher mono levels can reduce metal artifacts [16], but compromise image contrast and CNR, making in-stent restenosis difficult to detect (Figure 4). Iterative metal artifact reduction reconstruction (iMAR) can be effective in reducing metal artifacts from posterior spinal fixation [17], dental implants [18], or hip replacements [19]. However, for vascular stents, the use of iMAR can introduce additional artifacts that hinder the evaluation of the stent and related complications [20,21]. Therefore, this study did not explore the use of iMAR.

Sharper kernels on PCD-CT have been shown to reduce metal artifacts and improve visualization of the in-stent lumen [10,11]. In this study, both image contrast and subjective image quality improved with increasing kernel sharpness. This is because soft reconstruction kernels smooth the image through spatial interpolation, thereby increasing partial volume effects [22]. In contrast, sharper kernels emphasize higher spatial frequencies and thus suppress artifacts around the stent. Although image noise also increased, this effect can be considered negligible when evaluating images with high image contrast [23]. As mentioned by Arwed Elias Michael et al., an enhanced image contrast is more critical than image noise for detecting in-stent restenosis [13]. Notably, cardiac or respiratory motion artifacts may interact with the high-frequency noise introduced by sharp reconstruction kernels, thereby further degrading image quality. Although Qin et al. reported in a patient cohort that Bv72 was the optimal reconstruction kernel for the visualization of the stent strut and the in-stent lumen, its actual performance for the assessment of in-stent stenosis still requires further validation in clinical patient datasets [24].

A larger reconstruction matrix can improve image quality and diagnostic confidence in the assessment of peripheral arterial disease [25]. In this study, the 1024 matrix reconstruction was applied only on PCD-CT, and additionally reconstructed a 512 matrix for direct comparison with EID-CT. No significant difference in subjective image quality ratings was found between PCD-CT and EID-CT when using a 512 matrix. However, enlarging the matrix size in PCD-CT significantly improved subjective image quality for both medium-sharp and sharp kernels. According to the Nyquist theorem [26], the 512 matrix limits the maximum spatial frequency, potentially compromising part of the spatial resolution achievable with sharper kernels [27].

Due to the large number of reconstructions, only PCD-CT_SR-40keV_ was included in the subjective comparison with PCD-CT_UHR_ and EID-CT, as 40 keV VMIs achieved the highest image contrast and CNR among all VMIs. To better differentiate and rank image quality, we used pairwise comparative assessment rather than traditional Likert scoring. This approach yields more reliable subjective evaluation, which is valuable for optimization studies [8]. Subjective results showed a clear preference for VMI 40 keV, sharper reconstruction kernels, and larger matrix sizes. Notably, in mild stenosis, PCD-CT_SR_ demonstrated better restenosis visualization than PCD-CT_UHR_ and EID-CT, highlighting its potential for the early detection of mild in-stent restenosis. However, the detection of mild restenosis may also lead to more frequent reinterventions, and some of them may be unnecessary. Therefore, the clinical value and cost-effectiveness of such early detection require further investigation.

Image contrast and CNR are important objective image-quality metrics in CTA and can be used to reflect the visibility of the residual in-stent lumen, thereby influencing the detectability of in-stent stenosis. We found that both VMIs and kernels significantly affected image contrast and CNR, whereas the matrix mainly affected subjective image quality. Importantly, objective image-quality metrics do not fully reflect the actual capability for detecting in-stent stenosis. Therefore, future patient-based studies are needed to validate the optimized reconstruction settings using direct stenosis measurements and diagnostic performance metrics.

This study has several limitations. First, its ex vivo design inherently limits direct clinical generalizability, and prospective patient studies are needed. Second, although we included multiple restenosis severities, only one stent type was tested. Variations in stent diameter, stent materials and strut thickness can influence blooming and image quality. Thirdly, to allow for an objective evaluation of image quality, this study included only concentric in-stent stenoses. Nevertheless, eccentric stenoses are far more common in daily clinical practice. In addition, the diagnostic accuracy across various degrees of in-stent stenosis, occlusion, and patency stents need further investigation. Fourthly, this is a static phantom, and the pulsation of vessels in vivo may complicate the assessment of the stents. Fifthly, calcified plaque surrounding the was not included in the phantom, and its blooming artifacts may further impact the detection and evaluation of in-stent restenosis. Finally, we used clinical protocols on both CT scanners to reflect real-world practice, this resulted in differences in the radiation dose across scanners. Although higher doses were used for PCD-CT_UHR_ and EID-CT than for PCD-CT_SR_, the SR mode combined with VMI 40 keV and Bv72 still achieved the best image quality, suggesting potential for radiation dose reduction with PCD-CT. Future studies should further explore radiation dose and contrast agent reduction strategies using PCD-CT while validating the diagnostic performance of the optimized reconstruction parameters in patient cohorts.

## 5. Conclusions

PCD-CT in SR mode with VMI 40 keV, a sharp reconstruction kernel (Bv72), and 1024 matrix optimize the visualization of peripheral in-stent restenosis, which may serve as a reference protocol for post-EVT imaging follow-up. In addition, PCD-CT outperforms EID-CT in both quantitative and qualitative assessment of in-stent restenosis.

## Figures and Tables

**Table 1:** CT acquisition and reconstruction parameters of both systems.

	First Generation PCD-CT		Third Generation EID-CT
	Ultra-High Resolution	Standard Resolution	
Acquisition Parameters			
Tube voltage, kVp	120	120	120
Reference tube current time product, mAs	NA	NA	90
IQ Level	80	100	NA
Effective tube current time product, mAs	14	10	15
Rotation time, s	0.5	0.5	0.5
Pitch	0.85	0.8	0.6
CTDI vol, mGy	1.13	0.81	1.00
Reconstruction Parameters			
Matrix, pixels	1024 × 1024 & 512 × 512	1024 × 1024 & 512 × 512	512 × 512
FOV, mm	160	160	160
Slice thickness, mm	0.2	0.4	0.5
Increment, mm	0.2	0.3	0.3
Kernel	Bv40/Bv56/Bv72	Bv40/Bv56/Bv72	Bv40/Bv59
VMI	T3D	T3D, 40 kev, 55 kev, 70 kev, 85 kev, 100 kev	NA
Iterative reconstruction	QIR 3	QIR 3	ADMIRE 3

IQ, image quality; CTDI, Computed Tomography Dose Index; FOV, Field of view; VMI, virtual monoenergetic image; QIR, quantum iterative reconstruction algorithm; NA, Not Applicable.

**Table 2:** Quantitative analysis of different acquisition and reconstruction protocols.

	Acquisition and Reconstruction Protocols	Image Noise/HU	Image Contrast/HU	CNR
512 Matrix	EID-CT_Bv40_	30.7 ± 1.1	85 ± 23	2.78 ± 0.76
	EID-CT_Bv59_	115.0 ± 3.3	202 ± 57	1.76 ± 0.50
	PCD-CT_SR-Bv40-40keV_	34.3 ± 2.9	267 ± 54	7.77 ± 1.58
	PCD-CT_SR-Bv56-40keV_	79.4 ± 6.5	349 ± 92	4.39 ± 1.16
	PCD-CT_SR-Bv72-40keV_	160.3 ± 13.4	673 ± 116	4.20 ± 0.72
	PCD-CT_UHR-Bv40_	21.8 ± 1.4	112 ± 22	5.15 ± 1.01
	PCD-CT_UHR-Bv56_	46.2 ± 3.0	172 ± 33	3.72 ± 0.71
	PCD-CT_UHR-Bv72_	86.1 ± 5.9	245 ±46	2.85 ± 0.53
1024 Matrix	PCD-CT_SR-Bv40-40keV_	36.7 ± 1.1	274 ± 51	7.45 ± 1.38
	PCD-CT_SR-Bv56-40keV_	85.2 ± 2.1	380 ± 115	4.45 ± 1.35
	PCD-CT_SR-Bv72-40keV_	185.7 ± 4.4	741 ± 90	3.99 ± 0.48
	PCD-CT_UHR-Bv40_	22.0 ± 0.6	117 ± 19	5.34 ± 0.88
	PCD-CT_UHR-Bv56_	46.4 ± 0.7	170 ± 37	3.65 ± 0.80
	PCD-CT_UHR-Bv72_	84.0 ± 1.3	224 ± 39	2.67 ± 0.46
	*p*-value	<0.001	<0.001	<0.001

## Data Availability

The original contributions presented in this study are included in the article. Further inquiries can be directed to the corresponding author.

## Abbreviations

The following abbreviations are used in this manuscript:

CTA	Computed tomography angiography
CNR	Contrast-to-noise ratio
CTDI	Computed tomography dose index
EID-CT	Energy-integrated detector CT
FOV	Field of view
HU	Hounsfield units
IQ	Image quality
PCD-CT	Photon-counting detector CT
QIR	Quantum iterative reconstruction
ROI	Region of interest
SR	Standard resolution
UHR	Ultra-high resolution
VMI	Virtual monoenergetic image
